# Comparing the results of bisphosphonate use in clinical trials with actual practice: a case of apples and oranges?

**DOI:** 10.3390/curroncol13050018

**Published:** 2006-10

**Authors:** M.C. Gainford, G. Dranitsaris, W. Ooi, M. Vanhuyse, M. Clemons

**Keywords:** Bisphosphonates, breast cancer, bone metastases

## 1. INTRODUCTION

Bone metastases can be a devastating complication for any woman with breast cancer. Without treatment with a bisphosphonate, 50% of patients with metastases to bone will develop a skeletal related event (sre) such as hypercalcemia, spinal cord compression, or a pathological fracture or requirement for radiation or surgery (or both) for an impending fracture 1. These complications not only affect the patient’s quality of life, they can also directly affect mortality. Women with bone-only or bone-dominant disease have a median survival of 2–3 years, but an sre can shorten that median survival to just 12 months (fracture), 4 months (spinal cord compression), or 3 months (hypercalcemia of malignancy) 2,3. Delaying or reducing the occurrence of sres is therefore an important part of the care pathway for these patients.

In clinical practice, the commonly used bisphosphonates are clodronate, pamidronate, zoledronate, and ibandronate 4. Multiple randomized trials have confirmed their benefit, in conjunction with anticancer therapies such as chemotherapy or hormonal therapy, for significantly delaying or reducing sres 5. As a result, these agents have been rapidly integrated into routine practice; they are now a standard of care for systemic therapy of breast cancer patients with bony metastatic disease.

The American Society of Clinical Oncology recommends initiating treatment once radiologic evidence of bone destruction has been obtained, and once treatment is initiated, continuing it until it is no longer clinically relevant 6. A study of Canadian medical oncologists has confirmed that 90% of patients continue on bisphosphonates until death 7. Indeed, oncologists will rarely discontinue bisphosphonates even in the face of clear clinical deterioration.

This rapid implementation of clinical trial data into practice has major implications: financially on the health care system (because some of these agents have a substantial acquisition cost) and in terms of repeated clinic visits for intravenous treatment in patients entering the terminal phase of their illness, a time during which the derivation of any true benefit from continuing bisphosphonate therapy is unproven.

As is the case for all clinical trials, placebo-controlled studies of bisphosphonates have specific inclusion and exclusion criteria that could potentially limit generalizability. The patient population selected for a study are typically those who are most likely to gain maximum benefit from treatment. Several important differences exist between patients treated on study and those treated in non-study clinical practice.

First, the benefits of bisphosphonates are time-dependent, with maximum benefit gained after 6 months 8. Clinical trial populations have frequently been restricted by their inclusion and exclusion criteria to patients with an expected prognosis of at least 6 months 5.

Second, patients with bone-only disease are the ones who benefit most from treatment. Clinical trial populations therefore tend to contain mainly patients in whom bone-only disease predominates (Table I). In actual practice, patients with extensive visceral metastases and an expected prognosis of less than 6 months are routinely started on bisphosphonates and are continued on treatment despite repeated sres or bone progression 15. There is currently no data from randomized trials suggesting that continuing bisphosphonate therapy in patients with repeated sres while on a bisphosphonate produces continued patient benefit.

Third, the most frequently used primary endpoint in bisphosphonate trials is an analysis of sres. The definition of sre does not include variables that are important to patients, such as pain or immobility 5.

These differences between the two populations may limit the drawing of meaningful conclusions regarding the margin of benefit for bisphosphonate treatment outside of a trial setting.

## 2. COMPARISON OF NON-STUDY PATIENTS WITH CLINICAL TRIAL POPULATIONS

We previously reviewed the use of bisphosphonates in three Canadian centres 7. Briefly, we evaluated charts and electronic files of 190 breast cancer patients with bony metastases who commenced a bisphosphonate between January 2000 and December 2001 at the three centres. Patients already receiving bisphosphonates for osteoporosis or presenting with tumour-induced hypercalcemia were excluded from the review. We defined sres as pathologic bone fracture (vertebral or non-vertebral), spinal cord compression, surgery or radiation to bone, or development of hypercalcemia. To estimate the amount of benefit gained from bisphosphonate use in clinical practice, we compared our earlier non-study patients in terms of demographics and outcomes to the clinical trial populations (treatment arms) of the placebo-controlled trials.

Of the patients in our off-study population, 35% developed at least one sre (Table II). Except for a Japanese trial population in which only 31% of the patients were reported to have developed an sre 1, patients in the randomized trials were more likely to experience at least 1 event (range: 43%–52%). The mean number of sres per patient was 1.2 (range: 0–13 sres). Time to first sre was much shorter in the off-study population (Table II). Median time to the first event was estimated at 189 days. In contrast, patients treated with bisphosphonates in a randomized trial setting all had a median time to first event in excess of 300 days.

When the sres were analyzed by type, fewer fractures were reported in the off-study group: 12.6% versus 25%–36% (Table III). The frequency of other sre types—radiation, surgery, spinal cord compression, and hypercalcemia—were similar in the two groups.

## 3. DISCUSSION

Bisphosphonates have rapidly become a standard treatment for breast cancer patients with bone metastases, their benefit having been proven in multiple randomized trials 1,9–14. However, many uncertainties remain regarding their use, including the optimal duration and timing of treatment. Indeed it is even unclear which patients should be treated and which should not 5.

This review has already highlighted the three major differences between trial populations and patients treated in routine clinical practice. Only 29% of patients in our clinical practice population had bone-only disease, meaning that the overall group had an inherently poor prognosis. They were probably less likely to benefit from bisphosphonate treatment because they would likely not live long enough to gain the time-dependent benefits of treatment 8. They were sicker overall as reflected in the much shorter time to first sre.

Patients in our clinical population, most of whom had bone and visceral metastases, experienced fewer sres than did the randomized trial populations—with the exception of the Japanese trial patients 1. This finding supports previous work by Plunkett and colleagues, who reported that patients with bone-only disease at the time of diagnosis of skeletal metastases were 3–4 times more likely than those with bone and liver disease to develop pathologic long-bone fractures 16. The time to long-bone fracture was similar for all groups, but the least number of fractures occurred in the patients with concomitant visceral metastases. This finding reflects the shorter survival time for patients with visceral disease (5.5 months vs. 2.2 years). Therefore, patients with bone-only metastases are the ones who tend to live long enough to derive the time-dependent benefits of bisphosphonate treatment.

Evaluating the baseline sre hazard to determine its shape would be very interesting. Nonparametric approaches such as the Cox proportional hazards model and repeated events analysis do not permit a direct evaluation of the baseline hazard. Parametric alternatives such as the Weibull or Gompertz models should be considered, because these approaches would provide information on the hazard of sres over time.

The proportion of patients developing at least one sre was lower in the non-study population, but notably, although rates of episodes of radiation treatment, surgery, spinal cord compression, and hypercalcemia were comparable between the groups, significantly fewer fractures were reported in the non-study group than in the randomized trials group.

In clinical practice, patients do not tend to undergo serial imaging if they are asymptomatic. The events recorded are those that are clinically relevant. In contrast, patients enrolled in the randomized trials have serial X-rays performed at predefined intervals 11,12,14. This process captures symptomatic and asymptomatic events alike, thus ignoring pain, which is the most frequently reported symptom in daily practice 17. By performing serial imaging and including asymptomatic lesions—in particular, isolated rib or vertebral lesions—the trials may have overestimated the benefits of treatment. As a result, the data cannot be extrapolated to an estimate of the true clinical benefit for symptomatic lesions—the lesions that are important to patients.

The results from our study are consistent with previous published work 18. Liauw *et al.* recently reported that, in a cohort of 110 patients with stage iv breast carcinoma being treated with intravenous zoledronate or pamidronate, 30% developed a sre in the 12-month follow-up period. Only 9% of patients in that group experienced a fracture. Although the median time to first sre was much longer at 365 days, this result can perhaps be explained by the fact that 58% of the patients in that cohort had bone-only disease and therefore a better overall prognosis.

## 4. FUTURE DIRECTIONS

Not only do bisphosphonates use a significant proportion of the cancer drug budget 7, their prolonged use places a significant burden on patient and physician. Patients, many of whom are terminally ill, must return to their treatment centres on a monthly basis. Rationalizing the use of these agents is therefore important to patients, physicians, and policymakers. It may be sensible to restrict treatment to patients who would gain most from the time-dependent benefits of treatment. We need innovative strategies to identify the subset of patients with bone metastases who are at high risk for complications; we also need to improve our current methods of monitoring response to treatment.

Markers of bone resorption have shown great promise in both of these areas 19. Of all the markers under investigation, the N-terminal crosslinked type 1 collagen telopeptide (NTx) and C-terminal crosslinked type 1 collagen telopeptide (CTx) appear to be the most useful 20. In a study of 1824 patients with bone metastases treated with bisphosphonate, patients across all tumour types with the highest baseline levels of NTx had 2–3 times the risk of experiencing sres than did patients having low NTx levels (<50). Breast cancer patients had the highest risk [relative risk (rr): 2.96; *p* < 0.001). High and moderate NTx levels were both significantly correlated with risk of experiencing a first sre on study and of bone lesions progressing (*p* < 0.001). Overall, high NTx and moderate NTx correlated with a significantly greater relative risk of dying on study [rr: 4.8 (high) and 3.11 (moderate); *p* < 0.001] 21.

Dosing schedules of zoledronate are currently being evaluated in the bismark (Bisphosphonate Therapy Directed by Bone Resorption Markers) trial. Breast cancer patients with metastatic bone disease are randomized to receive either a fixed dose every 3–4 weeks or a modified schedule as determined by NTx level. This study will test the hypothesis that patients with normal levels of bone turnover (that is, in the range seen in individuals without metastatic breast cancer to bone) can receive less frequent treatment (R. Coleman, personal communication).

## 5. CONCLUSIONS

Given the inherent differences, as outlined here, between trial populations and patients treated in clinical practice, it is unlikely that we will ever know the true benefit of bisphosphonate treatment. What we can do is optimize our current use of these agents by means of innovative strategies. Bone markers offer the greatest hope of for optimization. Ongoing research should improve our understanding of these markers’ role in guiding treatment.

## Figures and Tables

**TABLE I:** Bone-only disease

*Bisphosphonate*	*Reference*	*Patients on study (*n*)*	*Patients with bone-only disease (%)*
Off-study population			
Clodronate PO/IV		190	29
Pamidronate IV			
Study populations			
Zoledronate 4 mg IV	Kohno *et al.*^1^	114	47 a
Ibandronate 50 mg PO	Tripathy *et al.*^9^	148	90 b
Ibandronate 6 mg IV	Body *et al.*^10^	154	69
Pamidronate 90 mg IV	Theriault *et al.*^11^	182	66
Pamidronate 90 mg IV	Hortobagyi *et al.*^12^	185	62
Pamidronate 90 mg IV	Hultborn *et al.*^13^	201	54
Clodronate 1600 mg	Paterson *et al.*^14^	85	32

a N. Kohno, personal communication.

b Bone plus soft tissue. D. Tripathy, personal communication.

PO = orally; IV = intravenously.

**TABLE II:** Patients and skeletal-related events (sres)

*Bisphosphonate*	*Reference*	*Patients experiencing at least 1sre(%)*	*Median time to firstsre(days)*
Off-study population			
Clodronate IV/PO		35	189
Pamidronate IV			
Study populations			
Ibandronate 50 mg PO	Tripathy *et al.*^10^	52	378
Ibandronate 6 mg IV	Body *et al.*^11^	51	354
Pamidronate 90 mg IV	Theriault *et al.*^12^	47	312
Pamidronate 90 mg IV	Hortobagyi *et al.*^13^	43	393
Pamidronate 90 mg IV	Hultborn *et al.*^14^	47	354

IV = intravenously; PO = orally.

**TABLE III:** Types of skeletal-related events (sres) at 12 months

	*Off-study population*	*Kohno* et al. *1*	*Theriault* et al. *11*	*Hortobagyi* et al. *12*
Bisphosphonate	Clodronate PO/IV Pamidronate 90 mg IV	Zoledronate 4 mg IV	Pamidronate 90 mg IV	Pamidronate 90 mg IV
Skeletal surveys	Clinical indication only	Before entry and 3, 6, 9, and 13 months	Before entry and 3, 6, 12 cycles	Before entry and 3, 6, 12 cycles
Type of sre (%)				
Fracture (total)	12.6	25	36	—
Vertebral			20	23
Non-vertebral			31	20
Radiation	19.5	8.8	21	19
Surgery	1.6	0	6	4
Spinal cord compression	1.6	3.5	2	2
Hypercalcemia	0	2.6	3	6

PO = orally; IV = intravenously.

## References

[1] Kohno N, Aogi K, Minami H (2005). Zoledronic acid significantly reduces skeletal complications compared with placebo in Japanese women with bone metastases from breast cancer: a randomized placebo controlled trial. J Clin Oncol.

[2] Coleman RE, Rubens RD (1987). The clinical course of bone metastases from breast cancer. Br J Cancer.

[3] Hill ME, Richards MA, Gregory WM, Smith P, Rubens RD (1993). Spinal cord compression in breast cancer: a review of 70 cases. Br J Cancer.

[4] Clemons M (2004). Should all breast cancer patients with symptomatic bone metastases be treated with bisphosphonates? The case in support. Clin Oncol (R Coll Radiol).

[5] Gainford MC, Dranitsaris G, Clemons M (2005). Recent developments in bisphosphonates for patients with metastatic breast cancer. BMJ.

[6] Hillner BE, Ingle JN, Chlebowski RT (2003). American Society of Clinical Oncology 2003 update on the role of bisphosphonates and bone health issues in women with breast cancer. J Clin Oncol.

[7] Clemons M, Enright K, Cesta A (2004). Do physicians follow systemic treatment and funding policy guidelines?. Can J Clin Pharmacol.

[8] Ross JR, Saunders Y, Edmonds PM, Patel S, Broadley KE, Johnston SR (2003). Systematic review on the role of bisphosphonates on skeletal morbidity in metastatic cancer. BMJ.

[9] Tripathy D, Lichinitzer Lazarev A (2004). Oral ibandronate for the treatment of metastatic bone disease in breast cancer: efficacy and safety results from a randomized double-blind, placebo-controlled trial. Ann Oncol.

[10] Body JJ, Diel IJ, Lichinitser MR (2003). Intravenous ibandronate reduces the incidence of skeletal complications in patients with breast cancer and bone metastases. Ann Oncol.

[11] Theriault RL, Lipton A, Hortobagyi GN (1999). Pamidronate reduces skeletal morbidity in women with advanced breast cancer and lytic bone lesions: a randomized, placebo-controlled trial. J Clin Oncol.

[12] Hortobagyi G, Theriault R, Porter L (1996). Efficacy of pamidronate in reducing skeletal complications in patients with breast cancer and lytic bone metastases. N Engl J Med.

[13] Hultborn R, Gundersen S, Ryden S (1999). Efficacy of pamidronate in breast cancer with bone metastases: a randomized, double-blind placebo controlled multi-center study. Anticancer Res.

[14] Paterson AH, Powles TJ, Kanis JA, McCloskey E, Hanson J, Ashley S (1993). Double blind controlled trial of oral clodronate in patients with bone metastases from breast cancer. J Clin Oncol.

[15] Verma S, Kerr–Cresswell D, Dranitsaris G (2004). Bisphosphonate use for the management of breast cancer patients with bone metastases. A survey of Canadian medical oncologists. Support Care Cancer.

[16] Plunkett TA, Smith P, Rubens RD (2000). Risk of complications from bone metastases in breast cancer: implications for management. Eur J Cancer.

[17] LoRusso P (2001). Analysis of skeletal events in breast cancer and response to therapy. Semin Oncol.

[18] Liauw W, Segelov E, Lih A, Dunleavy R, Links M, Ward R (2005). Off-trial evaluation of bisphosphonates in patients with meta-static breast cancer. BMC Cancer.

[19] Gainford MC, Dranitsaris G, Clemons M (2005). Systemic treatment of bone metastases from breast cancer—is it all that it’s cracked up to be?. J Clin Oncol.

[20] Clemons M, Cole DEC, Gainford MC (2006). Can bone markers guide more effective treatment of bone metastases from breast cancer?. Breast Cancer Res Treat.

[21] Coleman RE, Major P, Lipton A (2005). Predictive value of bone resorption and formation markers in cancer patients with bone metastases receiving the bisphosphonate zoledronic acid. J Clin Oncol.

